# Successful transcatheter mitral paravalvular leak closure complicated with stuck mechanical valve and device migration

**DOI:** 10.1093/ehjcr/ytae187

**Published:** 2024-04-12

**Authors:** Kenta Yoshida, Shunsuke Kubo, Takeshi Maruo, Kazushige Kadota

**Affiliations:** Department of Cardiology, Kurashiki Central Hospital, 1-1-1 Miwa, Kurashiki 710-8602, Japan; Department of Cardiology, Kurashiki Central Hospital, 1-1-1 Miwa, Kurashiki 710-8602, Japan; Department of Cardiology, Kurashiki Central Hospital, 1-1-1 Miwa, Kurashiki 710-8602, Japan; Department of Cardiology, Kurashiki Central Hospital, 1-1-1 Miwa, Kurashiki 710-8602, Japan

An 80-year-old man who underwent mitral and aortic mechanical valve replacement 27 years ago presented with haemolytic anaemia and heart failure. Transoesophageal echocardiogram (TEE) confirmed severe eccentric mitral paravalvular leak (PVL) (*Figure 1A*, Supplementary material online, *Video S1*). We performed transcatheter PVL closure using Amplatzer Vascular Plug II (AVP II, Abbott Vascular, IL).

**Figure 1:**
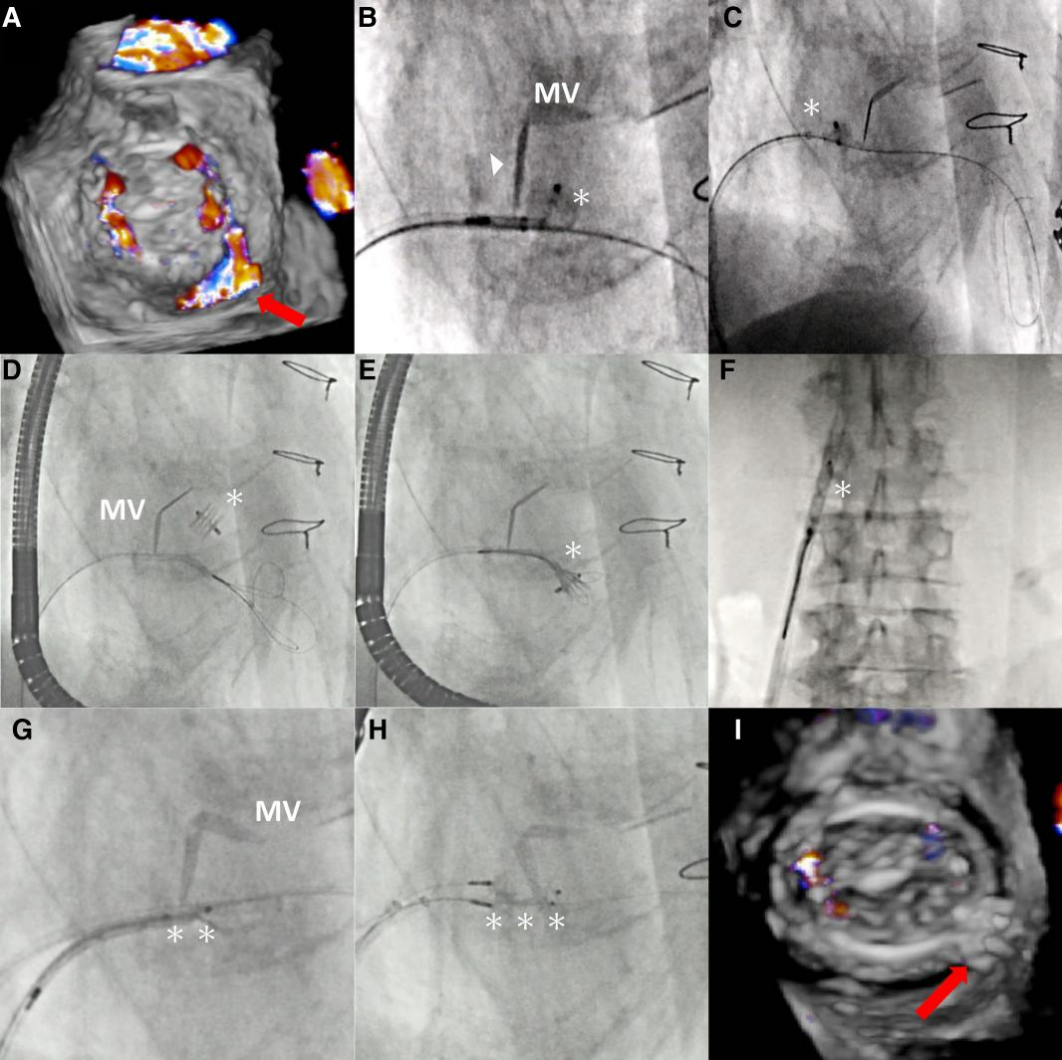
Three-dimensional colour images of the posteromedial paravalvular leak (PVL) (*A*: arrow). A 10 mm Amplatzer Vascular Plug (AVP) II (*) interfered with the mechanical mitral valve (*B*: arrowhead). Therefore, the device was deployed more proximally to avoid a stuck valve (*C*). However, the device accidentally dislodged and entered the left ventricle (*D*). After several attempts, the device was successfully snared and retrieved through the PVL (*E*, *F*). A 12 mm AVP II (**) caused a stuck mechanical valve (*G*). Simultaneously, the two downsized 8 mm AVP II (***) deployed also interfered the mechanical valve leaflet keeping it only partially opened (*H*). The PVL completely disappeared without mitral stenosis, and the devices were released (*I*).

Using the retrograde transseptal approach, a 10 mm AVP II was implanted in the PVL through a 6 Fr Destination sheath (Terumo, Japan). Because the device interfered with the mechanical valve leaflet, it was deployed more proximally (*Figure 1B* and *C*). After the PVL decreased to moderate, another device was tried to be added. When the sheath was re-advanced to the left ventricle (LV), the first device dislodged and moved to the LV (*Figure 1D*, Supplementary material online, *Video S2*). Then, a 7 Fr JR 4.0 guide catheter with an EN snare (Merit Medical, UT) was inserted into the LV through the PVL. After several attempts, the device was successfully snared and retrieved (*Figure 1E* and *F*). Using a 12 mm AVP II was difficult as it caused a stuck mechanical valve (*Figure 1G*). Therefore, two downsized 8 mm AVP II devices were deployed after the insertion of two 6 Fr Destination sheaths. The device’s distal disk interfered the mechanical valve leaflet, but it partially opened (*Figure 1H*, Supplementary material online, *Video S3*). With a mean mitral valve pressure of 4 mmHg, TEE didn’t display significant mitral stenosis, and the PVL had nearly disappeared (*Figure 1I*, Supplementary material online, *Video S4*). After confirming stability, the devices were released. The patient was uneventfully discharged 8 days following the procedure.

Device migration and stuck mechanical valve are important complications of PVL closure.^1^ Learning points of this case are follows: (1) the device migrated to the LV can be retrieved through the mitral PVL; and (2) when the device interferes the mechanical valve leaflet, the mechanical valve function can be preserved if the leaflet partially opens.

## Supplementary Material

ytae187_Supplementary_Data

## Data Availability

Data cannot be shared for ethical and privacy reasons.

## Supplementary material

**Consent:** The authors confirmed that written consent for submission and publication of this case report, including the images and associated text, has been obtained from the patient in accordance with COPE guidelines.
